# A New Self-Healing Hydrogel Containing hucMSC-Derived Exosomes Promotes Bone Regeneration

**DOI:** 10.3389/fbioe.2020.564731

**Published:** 2020-09-10

**Authors:** Li Wang, Jian Wang, Xiangbin Zhou, Jie Sun, Biao Zhu, Cuimi Duan, Peng Chen, Ximin Guo, Tong Zhang, Hongyan Guo

**Affiliations:** ^1^Graduate School, Jinzhou Medical University, Jinzhou, China; ^2^Key Laboratory for Biomechanics and Mechanobiology of Ministry of Education, School of Biological Science and Medical Engineering, Beihang University, Beijing, China; ^3^Medical Devices Control, National Institutes for Food and Drug Control, Beijng, China; ^4^Department of Stomatology, The Fifth Medical Center, Chinese PLA General Hospital, Beijing, China; ^5^Department of Stomatology, The First Medical Center, Chinese PLA General Hospital, Beijing, China; ^6^Department of Advanced Interdisciplinary Studies, Institute of Basic Medical Sciences and Tissue Engineering Research Center, Academy of Military Medical Sciences, Beijing, China; ^7^Department of Stomatology, The Third Medical Center, Chinese PLA General Hospital, Beijing, China

**Keywords:** human umbilical cord mesenchymal stem cells, exosomes, bone regeneration, self-healing hydrogel, bone graft material

## Abstract

**Background:**

Fractures are a medical disease with a high incidence, and about 5–10% of patients need bone transplantation to fill the defect. In this study, we aimed to synthesize a new type of coralline hydroxyapatite (CHA)/silk fibroin (SF)/glycol chitosan (GCS)/difunctionalized polyethylene glycol (DF-PEG) self-healing hydrogel and to evaluate the therapeutic effects of this novel self-healing hydrogel as a human umbilical cord mesenchymal stem cells (hucMSC)-derived exosome carrier on bone defects in SD rat.

**Methods:**

HucMSCs were isolated from fetal umbilical cord tissue and characterized by surface antigen analysis and pluripotent differentiation *in vitro*. The cell supernatant after ultracentrifugation was collected to isolate exosomes, which were characterized by transmission electron microscopy and western blot analysis. *In vitro* cell induction experiments were performed to observe the effects of hucMSC-derived exosomes on the biological behavior of mouse osteoblast progenitor cells (mOPCs) and human umbilical vein endothelial cells (HUVECs). The CHA/SF/GCS/DF-PEG hydrogels were prepared using DF-PEG as the gel factor and then structural and physical properties were characterized. HucMSCs-derived exosomes were added to the hydrogel and their effects were evaluated in SD rats with induced femoral condyle defect. These effects were analyzed by X-ray and micro-CT imaging and H&E, Masson and immunohistochemistry staining.

**Results:**

HucMSC-derived exosomes can promote osteogenic differentiation of mOPCs and promote the proliferation and migration of HUVECs. The CHA/SF/GCS/DF-PEG hydrogel has a high self-healing capacity, perfect surface morphology and the precipitated CHA crystals have a small size and low crystallinity similar to natural bone minerals. The MTT results showed that the hydrogel was non-toxic and have a good biocompatibility. The *in vivo* studies have shown that the hydrogel containing exosomes could effectively promote healing of rat bone defect. The histological analysis revealed more new bone tissue and morphogenetic protein 2 (BMP-2) in the hydrogel-exosome group. In addition, the hydrogel-exosome group had the highest microvessel density.

**Conclusion:**

A self-healing CHA/SF/GCS/DF-PEG hydrogel was successfully prepared. The hydrogel has excellent comprehensive properties and is expected to become a new type of bone graft material. This hydrogel has the effect of promoting bone repair, which is more significant after the addition of hucMSC-derived exosomes.

## Introduction

In recent years, more and more people are facing emerging musculoskeletal health problems due to osteoporosis, tumors and fractures (Zioupos et al., 1999). Currently, autogenous bone transplantation is a widely adopted method to solve this problem. However, autotransplantation requires at least two surgeries that can use cause complications and does not guarantee a positive result (Quan et al., 2017). An alternative is the allograft, but it has risks of suffering immunonological rejection, of causing infectious disease and lacks the capacity for osteogenesis. Therefore, the development of an ideal bone tissue engineering material that can integrate different compounds and incorporate growth factors to mimic the bone tissue microenvironment has become an increasingly research target in recent years (Dhand et al., 2016).

Recently, the application of exosomes derived from human umbilical cord mesenchymal stem cells (hucMSCs) in the field of bone regenerative medicine has been drawing significant attention. Zhang et al. (2017) found that hucMSC-derived exosomes can promote angiogenesis and fracture healing by up-regulating the expression of vascular endothelial growth factor and hypoxia-inducible factor-1α in rat models of femoral fracture. Li et al. (2019) evaluated hucMSC-derived exosomes in rats with steroid-induced femoral head necrosis (SNFH) and found that these exosomes can reduce the SNFH level they suggested that this mechanism may be related to the increase in VEGF and bone morphogenetic protein 2 (BMP-2) levels. Zhang et al. (2016) used hucMSC-derived exosomes to repair cartilage defects in rats and achieved excellent results.

A challenge for the application of functional and active substances, such as exosomes, to repair tissue damage is how to make them effectively gather in tissue defects and continue to exert effects. Stimulating endocytosis of exosomes by target cells is the only effective way to achieve their expected biological role. The production of exosomes in large quantities with high quality and purity is demanding, making clinical applications of exosomes more expensive. Extending the half-life of exosomes at the treatment site is essential for them to fully exert their therapeutic effect in low quantities. Liu et al. (2018) and Zhang Z. et al. (2018) found that relatively small exosomes encapsulated in a biodegradable hydrogel can still produce the expected therapeutic, even after weeks of application, because the hydrogel can prevent exosomes washout and maintains its local concentration. Besides, exosomes-loaded hydrogel can be applied directly to or near the treatment site, thereby making the delivery more targeted and reducing the exosomes dosage.

In the complex physiological environment, traditional hydrogels, such as gelatin, hyaluronic acid and fibrin glue, are incredibly prone to suffer damage by mechanical deformation that affects their mechanical properties. However, some hydrogels have self-healing characteristics. These characteristics allow, after being damaged, the hydrogel to repair itself in a short time, which results in an increase in the useful life and safety of this material. Based on the fact that biomedical hydrogels generally need to be used under physiological conditions, an ideal self-healing hydrogel material should adaptively achieve dynamic adjustment without resorting to external stimuli or energy input.

Multi responsive chitosan-based self-healing hydrogels constructed via dynamic imine bonds have been extensively studied (Zhang et al., 2011, 2012; Yang et al., 2012). After the double-end benzaldehyde-terminated telechelic polyethene glycol is used as a gel factor and mixed with a chitosan solution, an imine bond can be quickly formed at normal temperature to prepare a hydrogel. In these conditions, after the hydrogel is damaged by external force, it can obtain a quick self-healing without resorting to external conditions, avoiding the need for specific stimuli, such as pH, temperature or UV light. In addition, the difunctionalized polyethylene glycol (DF-PEG) gel factor is one of the few biocompatible, water-soluble and non-toxic polymer molecules that cause the entire hydrogel composite material to show excellent biocompatibility. The preparation process of this hydrogel material is simple and other functions can be conveniently added to the chitosan-DF-PEG hydrogel system by a straightforward method to obtain a new functional self-healing hydrogel to suit different applications.

**FIGURE 1:**
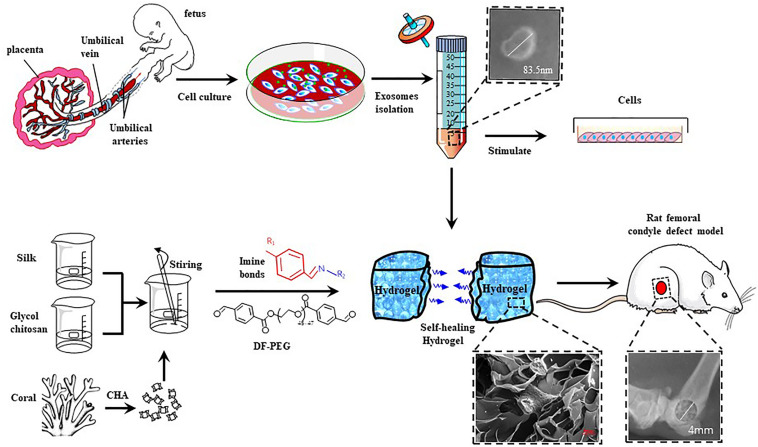
Schematic illustration of the isolation and characterization of hucMSC-derived exosomes and preparation of CHA/SF/GCS/DF-PEG hydrogel for testing in Sprague-Dawley (SD) rats with induced femoral condyle defect.

The use of chitosan alone can cause problems such as poor mechanical properties and unstable chemical properties (Azevedo et al., 2014). The emergence of the concept of composite materials promoted the widespread use of chitosan and silk fibroin (SF) composite scaffolds in bone tissue engineering research. Such composite scaffolds have excellent biocompatibility and osteoinduction but also have undesirable characteristics, such as rapid degradation, incompatibility with bone formation rate and poor mechanical properties (Li et al., 2018). Zeng et al. (2015) have demonstrated that the SF:GCS (2:3, v:v) is a good material for bone tissue engineering. The pore size of the scaffold was suitable for the growth of osteoblasts, and the rate of degradation was steady. This favors the early adhesion, growth and proliferation of MG-63 cells. In addition to good biocompatibility and satisfactory cell affinity, this material promotes the secretion of extracellular matrix materials by osteoblasts. Coralline hydroxyapatite (CHA) is a bone graft substitute derived from natural corals through hydrothermal exchange reactions (Georgiannos et al., 2015). CHA has excellent mechanical properties, high porosity, uniform pore size and has no blind holes and sensitization. It has a structure similar to cancellous bone and close to the inorganic formation of human bones. CHA can promote the proliferation, adhesion and osteogenic differentiation of mesenchymal stem cells (MSCs). The CHA surface can be chemically fused with the host tissue to form a tight bond. Subsequently, CHA is gradually absorbed and replaced by the original tissue, thus showing that CHA has excellent degradability and biocompatibility features (Brittberg et al., 1996). In addition, as the hydrogel has suitable plastic and adhesive properties, its incorporation reduces the fragility of CHA, preventing the migration of CHA particles. In the present work, we prepared a hydrogel containing CHA, SF, DF-PEG and glycol chitosan (GCS), in order to obtain a self-healing material with good mechanical properties and plasticity to meet the requirements to be used as material bone graft. We replaced chitosan with GCS based on a previous research (Yang et al., 2012) that showed that GCS has better solubility under physiological conditions. In addition, we evaluated whether this hydrogel can efficiently delivery hucMSC-derived exosomes directly to bone defect and then assess their effects in Sprague-Dawley (SD) rats with induced femoral condyle defect. The strategy used here for physical-chemical and biocompatibility assessment of CHA/SF/GCS/DF-PEG hydrogel and the bone tissue repair effects related to the hydrogel or the presence of exosomes is shown in Figure 1.

## Materials and Methods

### Animals

C57BL/6 suckling rat within 24 h of birth and 8-week-old male mice Sprague-Dawley (SD) rats were purchased from the Beijing Vital River Laboratory Animal Technology Co., Ltd. and maintained at the SPF Animal Laboratory of Academy of Military Sciences. All animal research followed the principles and procedures approved by the International Guiding Principles for Biomedical Research Involving Animals. Animal experimental ethical approval was obtained from the Academy of Military Sciences Animal Ethics Committee. All operations were performed under anesthesia and efforts were made to reduce animal suffering.

### Cell Culture

Human umbilical cord samples were obtained from healthy newborns after delivery under license from the parents of the babies and from the Institutional Review Board at the General Hospital of the Chinese People’s Liberation Army. hucMSCs primary culture was established according to a previous method (Song et al., 2016). Briefly, umbilical cord was collected at birth, stored on ice, and processed within 3 h of delivery. The umbilical cord was cut into 1∼2 mm^2^ pieces and rinsed with antibiotic-containing PBS. Wharton’s jelly was seeded in 100 mm culture plates and maintained in Minimum Essential Medium Eagle Alpha Modifications (α-MEM) (Gibco, Grand Island) containing 10% heat-inactivated fetal bovine serum (FBS) (Gibco) and 2 mmol/L l-glutamine (Gibco, Grand Island). After 2 days, the medium was replaced with a new one. After the cell culture reached 70–80% confluence, the cells were separated by treatment with TrypLE^TM^ Express (Gibco, Grand Island) and reseeded into a flask (75 cm^2^; Nunc, Denmark) at a density of 3000 cells per square centimeter (Nunc, Denmark). hucMSC of 2–6 passages were used in the study.

Isolation of mouse osteoblast progenitor cells (mOPCSs) was performed according to the adherent method (Li et al., 2016). Briefly, newborn mice were killed within 24 h after birth and calvaria was harvested, by meticulous removal of all non-osseous tissue, dura mater and the periosteum. The cranium was washed with PBS and digested with 0.1% collagenase A and 0.2% dispase to release osteoblasts. The bone fragments were spread on 100 mm culture plates and cultured in α-MEM supplemented with 10% FBS (Gibco, Grand Island, NE, United States), at 37°C in an atmosphere with 5% CO_2_. The medium was changed every 2 days and the second-generation cells were used for the present studies.

Human umbilical vein endothelial cells (HUVECs) were purchased from Clonetics^TM^ and maintained in an atmosphere of 5% CO_2_ at 37°C in α-MEM supplemented with 10% FBS.

### Analyzes of Surface Antigen and *in vitro* Multipotent Differentiation of hucMSCs

#### Flow Cytometric Analysis

hucMSCs surface antigen analysis was performed by flow cytometry. Briefly, 3 × 10^5^ hucMSCs were harvested by treatment with 0.25% trypsin-EDTA for 3 min to allow the cells to detach and washed twice with PBS. The cells were then incubated for 30 min at 4°C with a specific monoclonal antibody conjugated to either fluorescein and phycoerythrin (PE) in 200 μL PBS. Flow cytometry was performed to determine the expression of cell surface antigens using FACSCalibur (BD Biosciences). Antibodies for identification of CD31, CD44, CD45, CD73 surface markers (BD Biosciences) were used.

#### Osteogenic Differentiation

hucMSCs were seeded in a 6-well plate at a density of 1 × 10^5^ cells/well containing α-MEM medium supplemented with 10% FBS. After the cell fusion reached 60%, the osteogenic induction medium (α-MEM medium containing 10% FBS, 0.1 μM dexamethasone, 10 mM β-glycerol phosphate and 50 μg/ml ascorbic acid) was applied. The liquor was changed once every 3 days. After 14 days of induction, the cells were fixed and calcium accumulation was observed by Von Kossa’s staining.

#### Adipogenic Differentiation

hucMSCs were induced to adipogenic differentiation by culturing them in adipogenic differentiation medium (α-MEM medium containing 100 μM indomethacin, 10 μM insulin, 1 μM dexamethasone, and 0.5 mM 1-methyl-3-isobutylxanthine). After 14 days, adipogenesis was assessed by visualizing lipid droplet formation by Oil Red O staining.

#### Chondrogenic Differentiation

5 × 10^5^ hucMSCs were transferred to a 15 ml centrifuge tube and centrifuged at 800 *g* for 5 min at room temperature. The cellsform a pellet at the bottom of the centrifuge tube. Subsequently, the supernatant was gently removed and the pellet was transferred to chondrogenic induction medium (Cyagen Biosciences). The solution was changed every 3 days and the cells the cells were fixed on the 21st day. Paraffin-embedded tissue sections were Hematoxylin-Eosin (H&E) and immunohistochemical stained (Quan et al., 2017).

### Isolation and Identification of Exosomes

The exosomes were isolated from the cell culture medium as previously described (Zhang et al., 2019). Briefly, after hucMSCs reached 80% confluence, hucMSC of 2 to 6 passages were used in the study, the medium was replaced with fresh α-MEM without FBS and the cells were continued to be cultured for 24 h. Then, a new substitution with fresh α-MEM without FBS was performed and the cultivation continued for 48 h. Then, the culture was transferred to a conical tube and centrifuged at 16500 *g* for 20 min at 4°C to remove dead cells and cell debris. The supernatant was then filtered using a 0.22 μm cutoff filter to remove vesicles larger than that and transferred to a new tube that was subjected to ultracentrifugation at 120000 *g* for 70 min at 4°C in a SW32Ti rotor (Beckman Coulter, Brea, CA, United States) to pellet exosomes. The resulting supernatant was discarded and the pellet formed on the cone-shaped tubes wall was resuspended with distilled and deionized water and subjected to dialysis with distilled water for 3 days in a dialysis bag with a cutoff molecular weight of 500 Da (Millipore Inc.) to remove small protein fragments and ions. The dialysed solution was lyophilised to give a white flocculent exosome powder, which was then dissolved in phosphate buffer at a concentration of 1000 μg/mL and stored at −80°C for future use. The exosomes were analyzed by transmission electron microscopy (TEM), NanoSight (LM10, Malvern, United Kingdom) and Nanoparticle Tracking Analysis (NTA) software version 3.0 and their characteristic molecules, such as CD9 CD63, were identified by Western blotting.

### Impact of hucMSC-Derived Exosomes on mOPCs Osteogenic Differentiation

#### Alizarin Red Staining (ARS)

The formation of mineralized matrix nodules was determined by Alizarin Red staining. In summary, mOPCs were seeded in a 12-well plate at a density of 2.5 × 10^4^ cells/cm^2^. Three wells did not received hucMSC-derived exosomes (control group), three received the addition of 25 μg/ml hucMSC-derived exosomes and other three received 50 μg/ml hucMSC-derived exosomes. The cells were routinely cultured for 21 days and fixed with 4% paraformaldehyde solution for 10 min. Then, the cells were rinsed in distilled and deionized water and counterstained with 2% Alizarin Red (pH 4.0) for 5 min at 37°C. The cells were washed five times with PBS to reduce non-specific staining and images of them were obtained using a digital scanner. The Alizarin Red staining was dissolved in 10% (w/v) cetylpyridinium chloride at 37°C and the absorbance was measured at 570 nm for quantitative analysis.

#### Determination of Alkaline Phosphatase Activity

Alkaline phosphatase activity (ALP) staining kit (MesGenBiotech, China) was used to determine the alkaline phosphatase activity level in mOPCs under different conditions. The cells were treated in the same way as described for ARS. After the cells had been cultured for 7 days, the medium was discarded and the ALP staining kit was performed according to the manufacturer’s instructions. The staining results were semi-quantitatively analyzed using the IMAGE J software.

#### Impact of hucMSC-Derived Exosomes on HUVECs Proliferation

Three groups were created according to the concentration of hucMSC-derived exosomes (0, 25, and 50 μg/ml), as described above. P2 generation HUVECs were inoculated into 96-well plates at a density of 2000 cells/well. Each group was evaluated in quadruplicate. Then, the corresponding medium and exosome concentration from each group was added. The medium was changed every 2 days. The absorbance at 450 nm was measured by CCK-8 kit at 1, 3, 5, and 7 days of culture and the growth curve was drawn.

#### Scratch Wound Assay

The fused HUVECs layer was scratched on a 6-well plate using a P200 pipette tip to assess the impact of hucMSC-derived exosomes on HUVECs migration. After washing with PBS to remove loose cells, the experimental groups were divided with respect to the applied exosome concentration (0, 25, and 50 μg/ml), as described above. Each group was evaluated in triplicate. Then, 2 mL of the corresponding culture medium and exosome concentration from each group was added and the plates were incubated at 37°C. The images were acquired after 0, 8, and 16 h of incubation and then the reduction in the wound area was determined using Image-Pro Plus software (Media Cybernetics, Rockville, MD, United States). Cell counting was performed in three random fields.

### Preparation and Characterization of CHA/SF/GCS/DF-PEG Hydrogel

#### Synthesis of DF-PEG

DMAP (0.050 g), 4-formylbenzoic acid (0.98 g, 6.52 mmol) and PEG 2000 (3.26 g, 1.63 mmol) were dissolved in 100 mL of dry THF, followed by the addition of DCC (1.68 g, 8.15 mmol) under a nitrogen atmosphere. The mixture was stirred at 20°C for 24 h, and then, the white solid was filtered. The polymer was obtained after repeated dissolution in THF and precipitation in diethyl ether three times (Zhang et al., 2011).

#### Fabrication and Characterization of CHA Obtained by Hydrothermal Exchange of Nature Porous Coral

The corals were shattered and sieved to select particles of size 0.3–0.5 mm. These particles were washed 10 times with tap water and then soaked with sodium hypochlorite diluted 20x for 14 days. During this period, the liquid was changed 3–5 times and stirred. Then, the material was washed 3–5 times in boiling water, went trough 10 min of ultrasonic washing and another 10 washes with distilled and deionized water. The material dried overnight at 80°C. After complete drying, the material was weighed and applied to the reaction kettle with an equal mass of diammonium hydrogen phosphate. Then, a small amount of distilled and deionized water was added to the material under agitation and placed in the oven at 180°C for 10 h. After the reaction, the resulting material was transferred to a beaker, washed three times with distilled and deionized water, maintained in boiling water for 0.5 h and dried at 80°C. The following is the proposed exchange reaction for CHA described above:

10CaCO_3_ + 6(NH_4_)_2_HPO_4_ + 2H_2_O→Ca_10_(PO_4_)_6_(OH)_2_ + 6(NH_4_)_2_CO_3_ + 4H_2_CO_3_

The phase, morphology, element type and vibration modes of the CHA were analyzed by X-ray diffraction (XRD) (RINT PC1, Rigaku CO.), transmission electron microscopy (TEM), energy dispersive X-ray spectroscopy (EDS) (EMAX Energy, Horiba Ltd., Japan) and Fourier-transform infrared spectroscopy (FTIR) (Nicolet, AVATR360).

#### Hydrogel Preparation

The preparation of hydrogels refered to previous studies (Zhang et al., 2011; Zeng et al., 2015). Briefly, a 3% (w/w) solution of glycol chitosan (GCS) was prepared by dissolving specific amounts of chitosan in distilled and deionized water. The freeze-dried SF powder is dissolved in this chitosan solution to produce a 2% SF and 3% GCS mixed solution. Subsequently, CHA is added to the mixed solution to the surface of the liquid. 2.0 g of DF-PEG polymer were dissolved in 8.0 g of distilled and deionized water to obtain a 20% (w/w) DF-PEG solution. DF-PEG solution (1 mL) was added to GCS (2.8 g)/SF (1.86 g)/CHA (5 g) solution. After continuous stirring, the hydrogel was formed within 60 s at room temperature.

#### Hydrogel Characterization

##### SEM and EDS analyzes

The prepared CHA/SF/GCS/DF-PEG hydrogel wad pre-frozen at −20°C for 24 h. Then, the hydrogel was placed at −80°C for 24 h and lyophilized at −55°C until all the water was sublimated. An FE-SEM system equipped with an EDS system were used to examine Energy-dispersive EDS and the surface structure and morphology of the freeze-dried hydrogels.

##### XRD and FTIR analyzes

The freeze-dried CHA/SF/GCS/DF-PEG hydrogel was crushed and ground to below 300 mesh, and powder was taken for FTIR and XRD analysis to analyze the phase composition of the hydrogel sample.

##### Contact angle measurement

Surface wettability was detected by a measuring the contact angle on a JY-82A device (Dingsheng Testing Instrument Co. Ltd., China). A droplet of deionized water was placed on the surface of a sample and contact angle was measured at 0, 5, and 10 s. A 5% gelatin hydrogel was used as a control and at least six samples were analyzed for each state.

##### Self-healing experiment

The hydrogel was cut into six pieces and re-spliced. Thirty μl of PBS was added dropwise to the splicing place to moisten the surface. After splicing, it was placed at 37°C to observe the self-healing of the hydrogel and record pictures.

##### MTT assay for cytotoxicity

Generation P2 mOPCs were resuscitated and applied into a cell suspension with a cell concentration of 2 × 10^7^L^–1^. The cells were seeded into a 96-well plate with 3000 cells per well. After 24 h of standard culture, the medium was aspirated. Then, 300 μl of hydrogel extract were added to each well of the experimental group. This hydrogel extract is composed of CHA/SF/GCS/DF-PEG hydrogel immersed in an equal volume of α-MEM medium containing 10% FBS and incubated at 37°C for 24 h. In the control group, 300 μl of α-MEM medium containing 10% FBS without hydrogel was added to each well. The solutions (medium or medium + hydrogel) was changed every two days. The absorbance at 492 nm wavelength was measured in a microplate reader 1, 3, 5, and 7 days after hydrogel extract addition. Each time point collected was measured in quadruplicate.

##### Sustained release properties of CHA/SF/GCS/DF-PEG hydrogel

The sustained release properties of the CHA/SF/GCS/DF-PEG hydrogel were evaluated and compared with gelatin hydrogel. The same procedure as described above was performed to prepare hydrogel, but we used PBS containing exosomes to dissolve GCS instead of using PBS. The release of exosomes from gelatin hydrogel and CHA/SF/GCS/DF-PEG hydrogel was tested by BCA protein assay. Briefly, 100 μL of a 1 μg/μl exosome solution was used to prepare the hydrogel system. Then, the hydrogel system was placed in PBS at 37°C. The supernatants were collected continuously for 30 days to calculate the amount of exosome\release and to draw a release curve.

##### Evaluation of biological characteristics of the three-dimensional culture of mOPCs

The survival status of mOPCs in CHA/SF/GCS/DF-PEG hydrogels was evaluated using the fluorescein diacetate/propidium iodide (FDA/PI) staining method. The mPOBs were suspended in α-MEM medium, mixed with CHA/SF/GCS dissolved in α-MEM medium and then transferred to a Petri dish. Finally, DF-PEG dissolved in α-MEM medium was drop into the Petri dish and mixed gently to induce gel formation. After gel formation, α-MEM medium is added, followed by conventional culture. Six samples were prepared and three were taken on the third day. The condition of the cells was observed under an inverted microscope. The samples were stained with the FDA/PI reagent and quickly moved to a fluorescence microscope to observe and record pictures. The remaining three samples were cultured for 10 days to assess the effect of degradation on the cells.

### *In vivo* Evaluation of Bone Regeneration

#### Animal Surgical Procedure

Twelve hours before surgery, fifty-four rats were fasted and randomly divided into three experimental groups: (A) negative control group (control); (B) CHA/SF/GCS/DF-PEG hydrogel group (hydrogel) and; (C) CHA/SF/GCS/DF-PEG hydrogel with exosomes group (hydrogel-exosomes). The rats were submitted to general anesthesia and, with adequate aseptic precautions, a 1.5 cm longitudinal incision was made in the center of the accessible bone bulge on the outside of each leg in the femoral condyle. Subcutaneous tissue, fascia, muscle and periosteum were carefully dissected to expose the underlying bone. Adjust the parameters of dental micromotor. The bone defect was induced by creating a 4 mm depth hole in the underlying cancellous bone using a 4 mm ring bone drill. During the cirurgy, cold physiological saline solution was used constantly to wash the surgery site and avoid excessive heat production and tissue necrosis. Each experimental group was processed as follows: (A) control group: 100 μl of PBS were added to the bone defect site of the rats, and the PBS overflowing the defect area was dipped dry with gauze. (B) hydrogel group: Added 3 μg GCS and 2 μg SF to 100 μl PBS, fully dissolve, CHA was added to the mixed solution to the surface of the liquid, the quality was about 5.4 μg, after mixing, added 5 μl of 20% DF-PEG solution, stired thoroughly. Through a needleless 1 ml syringe, the hydrogel was implanted tightly in the holes and applied in their surrounding area before hydrogel forming, in order to guarantee a complete filling of the induced defect. After the hydrogel was formed, the hydrogel outside the bone defect area was removed with a sharp scalpel. (C) Hydrogel-exosome group: Considering the application requirements of exosomes, in order to do achieve an effective dose response, at least 10∼100 μg of exosomes is required, and the bone defect volume was about 0.05 ml, so we will make the concentration of 1000 μg/ml exosome solution instead of PBS to prepare exosome-loaded CHA/SF/GCS/DF-PEG hydrogel for bone defect treatment, the theoretical value of exosomes in the defect area was 50 μg. The skins were sutured with interrupted 2/0 Ethilon R sutures. The rats were housed individually and received an intraperitoneal injection of penicillin. Regular checks of their conditions were performed in the first three days after surgery.

#### Retrieval of Specimens

The rats were euthanized with an excess of barbiturate (1 mg/kg) at 30, 60, and 90 days after implantating the specimens. The femoral condyles and their surrounding area were removed to assess possible potential inflammatory reactions related to polymer use in the body and for photographic records. The specimens were collected and fixed in 10% neutral-buffered formalin for 12 h.

#### Micro-CT and X-ray Imaging Analyzes

Micro-computed tomography (Micro-CT; Germany, Bruker) was performed using a 280 μA source current, a source voltage of 90 kV and an exposure time of 550 ms. The scanning was performed with the same calibration parameters, the sagittal and axial planes of each defect area were reconstructed using NRecon software, and then three-dimensional analysis was performed. Bone mineral content (BMC) and new bone volume (BV/TV%) were calculated to quantify new mineralized tissue. X-ray images were taken in the Department of Stomatology, General Hospital of People’s Liberation Army Third Medical Center, 30, 60, and 90 days after retrieval of specimens.

#### Histological Analysis

Histological analysis was performed to assess bone healing in induced defects. The samples were decalcified using 10% ethylenediaminetetraacetic acid (EDTA) buffer solution for 30 days, washed three times with PBS, dehydrated and then embedded in paraffin. Slices of 3–5 μm were subjected to H&E and Masson’s trichrome staining. The stained sections were observed under compound microscope (Nikon, Japan). The images were captured using a DS-U3 imaging system (Nikon).

#### Immunohistochemical Analysis

BMP-2 and CD34 staining were used to determine BMP-2 deposition and angiogenesis in bone defect areas in each experimental group. The sections were treated with antigen retrieval and then incubated with primary antibody (rabbit anti-rat, Abcam) at 4°C overnight. Subsequently, antibody binding in the tissue sections was observed by incubation with DAB substrate. The CD34 and BMP-2 staining were observed under compound microscope. For each slide, Image-Pro Plus 6.0 software was used for semi-quantitative analysis of staining results in four random fields with 400 × magnification.

### Statistical Analysis

All experiments were performed at least in triplicate. All the data are presented as the mean ± standard deviation (SD). Statistical analyzes were performed by Student’s *t*-test using SPSS 19.0 software (SPSS Inc., United States). *P*-values < 0.05 were considered statistically significant.

## Results

### Characterization and *in vitro* Multipotent Differentiation of hucMSCs

**FIGURE 2:**
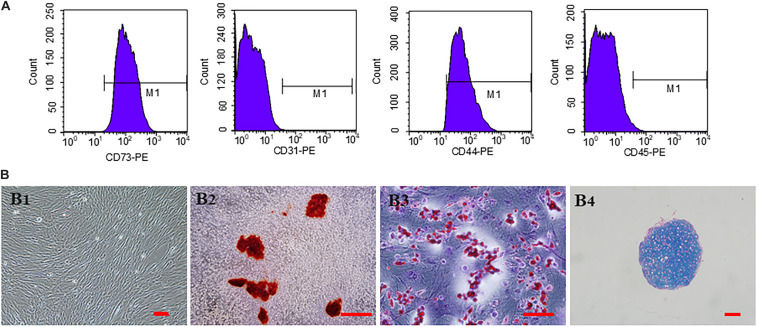
Characterization and *in vitro* multipotent differentiation of hucMSCs. **(A)** Flow cytometric analysis of surface markers presented by hucMSCs. **(B)** Representative images of third generation hucMSCs **(B1)**. Representative images of osteogenesis **(B2)**, adipogenesis **(B3)**, and chondrogenesis **(B4)** of hucMSCs after Allizerin Red, Oil Red O and Alcian blue staining, respectively. Scale bar: 100 μm.

The third generation hucMSCs exhibited a spindle-like morphology *in vitro* (Figure 2B1). The results of trilineage differentiation experiments proved the pluripotency of hucMSCs (Figures 2B2–B4). Flow cytometry analysis of surface markers revealed that the cultivated hucMSCs were positive for MSC markers CD44 (98.7%) and CD73 (100%), while they were negative for hematopoietic stem cell markers CD31 (0.059%), CD45 (1.06%) (Figure 2A). These results were consistent with previous studies (Dhand et al., 2016).

### Characterization of hucMSC-Derived Exosomes

**FIGURE 3:**
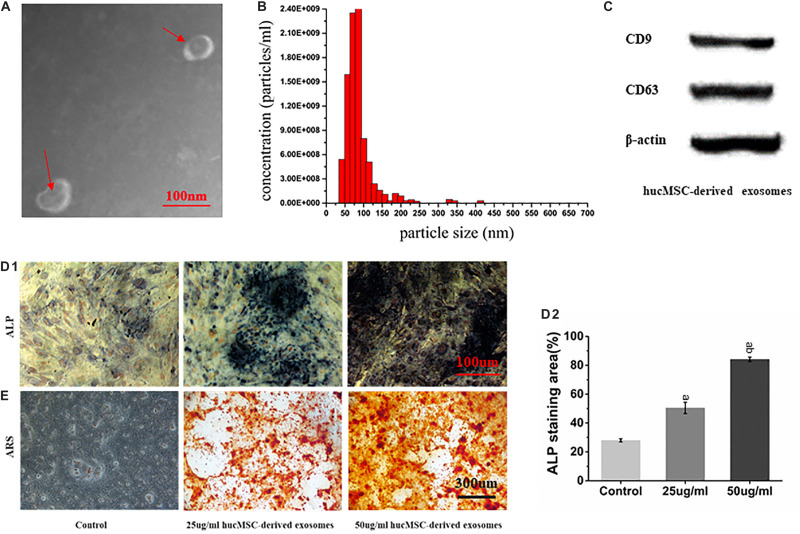
Characterization of hucMSC-derived exosomes. **(A)** Representative images of the morphology of hucMSC-derived exosomes by transmission electron microscopy. The red arrow indicates exosomes. Scale bar: 100 nm; **(B)** Particle size distribution of exosomes detected by NanoSight and the mean diameter is 90 nm; **(C)** Western blot identification of exosomes surface markers; **(D1)** ALP staining results from stimulation of hucMSC-derived exosomes on mOPCs after 7 days of culture. Scale bar: 100 μm; **(D2)** Alkaline phosphatase activity (ALP) staining area (%); **(E)** Alizarin Red Staining (ARS) results from stimulation of hucMSC-derived exosomes on mOPCs after 21 days of culture. Scale bar: 300 μm.

hucMSC-derived exosomes were successfully collected, showing a spherical structure, as observed by TEM (Figure 3A) and a diameter of about 90 nm (Figure 3B). Western blot results showed that hucMSC-derived exosomes expressed the characteristic surface markers CD9 and CD63 (Figure 3C).

### hucMSC-Derived Exosomes Promote mOPCs Osteogenic Differentiation

ARS results showed that, after 21 days of mOPCs culture, the hucMSC-derived exosomes group could secrete more mineralized matrix and a greater number of calcified nodules was formed than in the control group (Figure 3E). The semi-quantitative analysis results showed that the control group presented an Abs_570 nm_ of 0.421 ± 0.10 while the groups that received 25 and 50 μg/ml of hucMSC-derived exosome presented higher Abs_570__nm_ values of 1.852 ± 0.24 and 2.37 ± 0.22, respectively. The formation of calcified nodules increased with increasing exosomal concentration and also showed a significant dose dependence.

ALP staining results showed that, after mOPCs had been cultured for 7 days, hucMSC-derived exosome group showed a large amount of brown cobalt sulfide particles in the cytoplasm (Figure 3D1). The semi-quantitative analysis results of ALP staining using the IMAGE J software indicated that the groups that received 25 and 50 μg/ml of hucMSC-derived exosomes groups showed significant alkaline phosphatase activity higher than the control group (*P* < 0.05) (Figure 3D2). In addition, alkaline phosphatase activity enhanced with the increasing exosomal concentration, indicating a significant dose dependence.

### Characterization of the CHA/SF/GCS/DF-PEG Hydrogel

**FIGURE 4:**
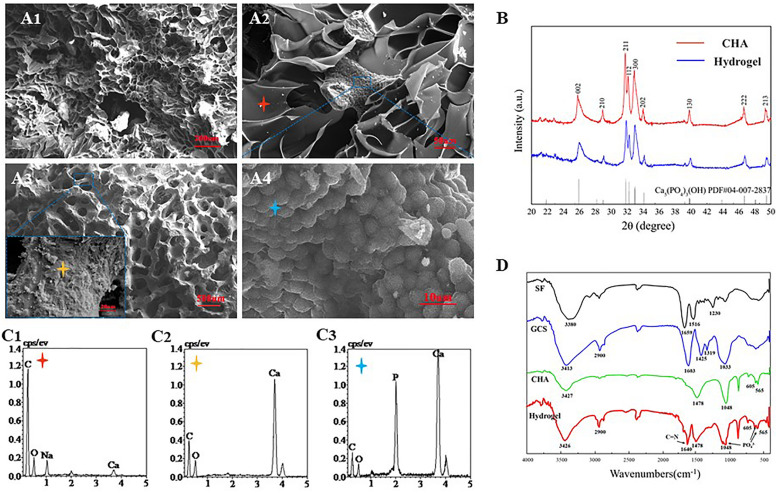
Scanning electron microscopy (SEM) and X-ray diffraction (EDS) analyzes of CHA/SF/GCS/DF-PEG hydrogel. **(A1)** 200× (Scale bar: 20 μm) and **(A2)** 500× magnification (Scale bar: 50 μm) of SEM images of CHA/SF/GCS/DF-PEG hydrogel. **(A3)** Surface morphology of corals (100× and 5.00K× magnification; scale bar: 500 and 20 μm, respectively). **(A4)** Surface morphology of hydrothermally converted sea coral (5.00K× magnification; scale bar: 10 μm). **(B)** X-ray diffraction (XRD) pattern of CHA and CHA/SF/GCS/DF-PEG hydrogel. **(C1–C3)** Red, yellow and blue stars correspond to the EDS analysis results, respectively. **(D)** Fourier-transform infrared (FTIR) spectra of SF, GCS, CHA and CHA/SF/GCS/DF-PEG hydrogel.

Fourier-transform infrared (FTIR) spectrum results showed that the SF exhibits characteristical lyamide bands I (C = O stretching, around 1659 cm^–1^); lyamide bands II (NH deformation, around 1516 cm^–1^), and lyamide bands III (O–C–N bending, around 1230 cm^–1^). GCS presents absorption bands at 2900 cm^–1^ (–CH_2_), 1603 cm^–1^ (−NH_2_), 1425 cm^–1^ [–COOH), 1319 cm^–1^ (amide III) and 1033 cm^–1^ (C–O)] (Yamaguchi et al., 2001). The characteristic absorption bands of CHA at the peaks of 1048, 605, and 565 cm^–1^ should be the result of the deformation vibrations of PO_4_^3–^. In relation to the carbonate group, a peak at 1478 cm^–1^ is observed in the CHA spectra (Figure 4B). These data were consistent with previous literature reports (Nandi et al., 2015). Some authors have studied the interaction between organic elements and HA in SF/HA nanocomposites (Wang and Li, 2006). They proposed that this interaction occurs between negative charges of the functional groups of the inorganic matrix and Ca^2+^ ions. In the FITR spectrum of lyophilized CHA/SF/GCS/DF-PEG hydrogel, a new peak at 1640 cm^–1^ was observed. This data indicates that the imine bond formation occurred in hydrogel network since it is the result of the stretching vibration of C = N of imine bond (Zhang Z. et al., 2018). The peaks of the composite hydrogels at 1048 cm^–1^ and 605 cm^–1^ in accordance with PO_4_^3–^ signal confirm the presence of well-crystallized CHA (Figure 4B).

CHA/SF/GCS/DF-PEG hydrogel and pure CHA shown similar X-ray diffraction (XRD) patterns (Figure 4D). These data show that the involvement of GCS and SF does not change the crystallographic structure of CHA in the CHA/SF/GCS/DF-PEG hydrogel. CHA and hydrogel showed obvious spectral broadening and peak overlap, indicating that precipitated CHA crystals have a small size and low crystallinity similar to natural bone minerals (Zhang et al., 2015).

### CHA/SF/GCS/DF-PEG Hydrogel Morphology

Analysis of SEM images of CHA/SF/GCS/DF-PEG hydrogel shows that their pores have mostly a circular ellipse shape of the same size and that they are well connected to each other. These images also show that the thickness of the pore walls is uniform, the surface is smooth and the porosity is 91.24 ± 12.85% (Figures 4A1,A2). SEM images of coral indicated the coral was complete without cracking (Figure 4A2). Finally, SEM images also showed that a large number of spherical crystals were attached to the surface of the CHA after hydrothermal reaction treatment of the coral (Figure 4A4). EDS analysis revealed that the calcium carbonate on the surface of the coral was converted into calcium phosphate (Figures 4C1–C3). These data indicated that CHA was successfully prepared.

### CHA/SF/GCS/DF-PEG Mechanical, Self-Healing, Sustained Release Rate and Hydrophilic Properties

**FIGURE 5:**
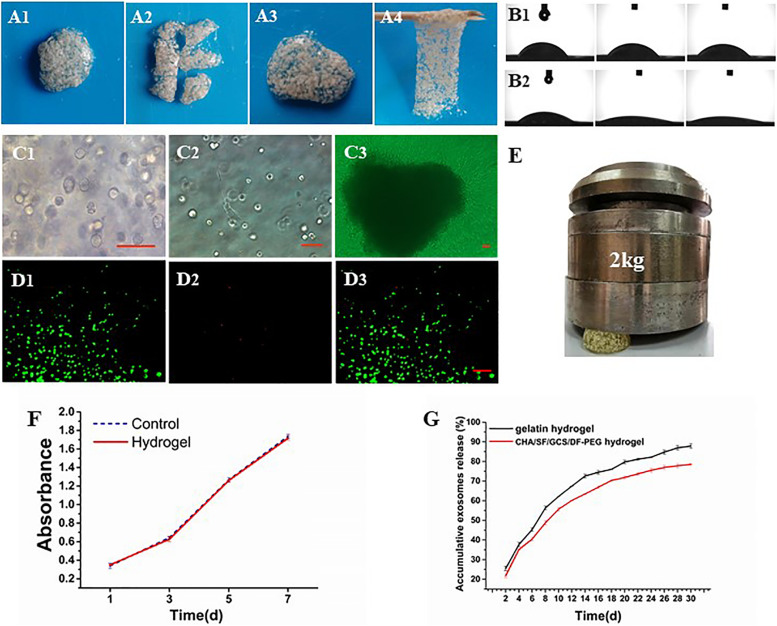
Characterization of the CHA/SF/GCS/DF-PEG hydrogel. **(A1–A4)** Self-healing evaluation. **(B)** Contact angle experiment, being **(B1)** the gelatin hydrogel and **(B2)** the CHA/SF/GCS/DF-PEG hydrogel. **(C)** Observation of the growth status of mOPCs in CHA/SF/GCS/DF-PEG hydrogel using an optical microscope (panels **(C1–C3)** represent the mitotic phase of the cell, the elongation of cells at the bottom of the plate and the release of cells resulting from hydrogel degradation, respectively). **(D)** LIVE/DEAD staining, **(D1)** viable cells: green, **(D2)** dead cells: red, **(D3)** merge of viable cells and dead cells. **(E)** Images of the CHA/SF/GCS/DF-PEG hydrogel being compressed by a weight of 2 kg. **(F)** MTT assay for cytotoxicity evaluation of hydrogel. **(G)** Release profiles of exosomes from the gelatin hydrogel and CHA/SF/GCS/DF-PEG hydrogels.

The compression of the CHA/SF/GCS/DF-PEG hydrogel by a weight of 2 kg shows that it is capable of supporting this weight without major deformations (Figure 5E), indicating that the hydrogel has a good mechanical resistance. After cutting the CHA/SF/GCS/DF-PEG hydrogel, it can fuse again at 37°C within half an hour without external stimulation and with the complete disappearance of the incision lines (Figures 5A1–A4). This self-healing hydrogel can be repeatedly stretched without breaking. In addition, even after being pulled hard, it does not necessarily break where it was previously incised. These observations indicate that the CHA/SF/GCS/DF-PEG hydrogel is fully self-healing. The contact angle of water droplets in the CHA/SF/GCS/DF-PEG hydrogel 0, 5, and 10 s after placing these droplets on its surface is much smaller than that observed in the gelatin hydrogel (Figures 5B1,B2). The exosome release analysis revealed that CHA/SF/GCS/DF-PEG hydrogels had a relatively slower release effect on exosomes than gelatin hydrogels. After 30 days of continuous measurement, 78.22 ± 0.36% of the exosomes were released from the CHA/SF/GCS/DF-PEG hydrogels while the number of gelatin hydrogels was 87.07 ± 1.12% (*P* < 0.05, Figure 5G). These data indicate that the CHA/SF/GCS/DF-PEG hydrogel has excellent hydrophilicity.

### Biocompatibility of CHA/SF/GCS/DF-PEG Hydrogel in mOPCs

The cytotoxicity of the CHA/SF/GCS/DF-PEG hydrogel in mOPCs was detected by the MTT assay. At the same detection time, there were no differences in absorbance at 492 nm between the CHA/SF/GCS/DF-PEG hydrogel experimental group and the control group (*P* > 0.05; Figure 5F). Thus, it can be seen that the CHA/SF/GCS/DF-PEG hydrogel has no significant effect on the proliferation of mOPCs (Figure 5F).

### Three-Dimensional Culture of mOPCs in CHA/SF/GCS/DF-PEG Hydrogels

The mOPC three-dimensional cultured cells in CHA/SF/GCS/DF-PEG hydrogel are mostly round or oval and have distinct mitotic phases (Figure 5C1), although extended spindle cells can be seen at the bottom of the well plate (Figure 5C2). On the 7th day of culture, after the hydrogel degradation, a large number of cells crawled of its surroundings. These cells were spindle-shaped, the body was full, and the proliferation rate was fast (Figure 5C3). LIVE/DEAD staining of mOPCs in CHA/SF/GCS/DF-PEG hydrogel was visualized under a fluorescence microscope. Living cells showed green fluorescence while dead cells showed red. On the 3rd day of culture, the mOPCs in the hydrogel were spherical. The staining results showed that the green fluorescence (365.33 ± 13.15) was significantly stronger than the red fluorescence (30.22 ± 2.00) and that the survival rate was greater than 90% (Figures 5D1–D3). These data indicate that mOPCs showed good survival in a gel environment.

### CHA/SF/GCS/DF-PEG Hydrogel Associated With hucMSC-Derived Exosomes Accelerates New Bone Formation and Improves Osseointegration

#### Gross Specimen Observation

**FIGURE 6:**
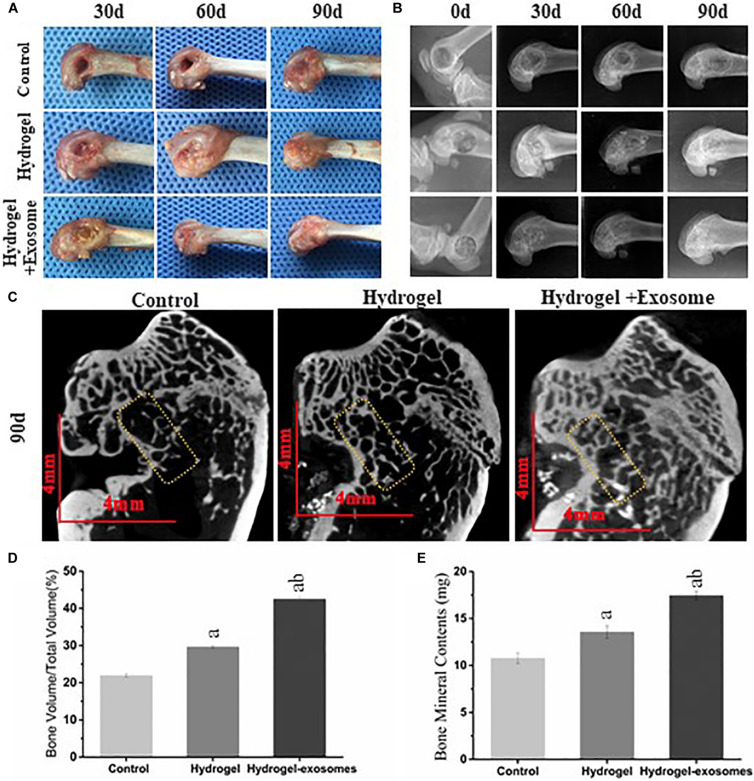
Gross observation and X-ray and Micro-CT imaging results of the bone defects 0, 30, and 90 days post-implantation of CHA/SF/GCS/DF-PEG hydrogel or CHA/SF/GCS/DF-PEG hydrogel associated with hucMSC-derived exosomes. **(A)** Representative images of defect surface tissue regeneration after 0, 30 and 90 days of hydrogel preparations implantation. Evaluation of new bone by X-ray **(B)** and Micro-CT **(C)** images after 0, 30, and 90 days of implantation (Scale bar = 4 mm). Values of new bone volume (BV/TV%, panel **D**) and bone mineral contents (BMC, panel **E**) calculated from the Micro-CT data analysis. The yellow rectangle represents the bone content measurement area. a, *P* < 0.05 compared to the control group; b, *P* < 0.05 compared to the hydrogel group.

Upon collecting the samples, no apparent tissue hyperplasia and suppuration were found in the area surrounding the induced bone defect. The muscle layers were cut layer by layer (Figure 6A). In the hydrogel and hydrogel-exosomes experimental groups, there was no apparent immune rejection reaction between the surrounding tissues and the CHA/SF/GCS/DF-PEG composite material. In addition, there was no osteonecrosis. Furthermore, 30 and 60 days post-implantation of CHA/SF/GCS/DF-PEG hydrogel or CHA/SF/GCS/DF-PEG hydrogel associated with hucMSC-derived exosomes, the cavity in the bone defect area was still clearly visible in the control group. On the other hand, in the hydrogel and hydrogel-exosomes experimental groups, the CHA/SF/GCS/DF-PEG composite was tightly combined with the tissue surrounding of the bone defect, which surface had been covered by a layer of soft tissue (Figure 6A). The hydrogel-exosomes group was more densely packed than the hydrogel group. After 90 days of implantation, the bone defect in the control group was still visible, but superficial, with shallow cavities. In the hydrogel and hydrogel-exosomes groups, in turn, the bone defects had healed and there was no noticeable difference with the naked eye (Figure 6A).

#### X-ray and Micro-CT Imaging Results

The X-ray image results on day 0 after the implantation of CHA/SF/GCS/DF-PEG hydrogel or CHA/SF/GCS/DF-PEG hydrogel associated with hucMSC-derived exosomes, show that the femoral condyle model was successfully prepared, the bone walls of the bone defect were intact and no fractures were observed (Figure 6B). Moreover, the hydrogel and hydrogel-exosomes experimental groups were shown filled with material to prevent projection and no overflowing material was found. A clear boundary could be seen between the bone and the surrounding bone tissue. After 30 days, the boundaries between the bone defect and the surrounding tissues are still clearly visible in the hydrogel and hydrogel-exosomes groups. The hydrogel-exosomes group showed more blurred than the hydrogel group, while the control group had apparent voids (Figure 6B). After 60 days, the hydrogel-exosomes group had a weaker boundary, almost disappeared, between the bone defect and the surrounding bone tissue than the hydrogel group. In the control group, the voids decreased slightly, but were still very visible (Figure 6B). Finally, after 90 days, the bone cortex and surrounding bone tissue were completely closed and covered in the hydrogel-exosomes group. On the other hand, the bone cortex in the hydrogel group was not completely covered and apparent voids were still visible in the control group (Figure 6B).

Micro-CT images of all groups 90 days post-implantation of CHA/SF/GCS/DF-PEG hydrogel or CHA/SF/GCS/DF-PEG hydrogel associated with hucMSC-derived exosomes (Figure 6C) show that observed the bone regeneration is consistent with that indicated by the X-ray image analysis: bone regeneration level of hydrogel-exosomes group > hydrogel group > control group. After 90 days, the hydrogel-exosomes group presented a new bone and trabeculae arranged neatly and densely as well as a completely closed cortical bone with a specific thickness On the other hand, in the hydrogel group, it can be seen that the trabeculae of the new bone were disordered and diluted and that the cortical bone was thin. Finally, in the control group, the number of trabeculae of the new bone was small, its arrangement was disordered and the surrounding bone was hardened (Figure 6C). The values of new bone volume (BV/TV%) and bone mineral contents (BMC) at the bone defect site were calculated from the micro-CT data analysis after 90 days of implantation (Figures 6D,E). The results showed that the BV/TV% and BMC values of the hydrogel-exosomes group were significantly higher than that of the hydrogel group. The hydrogel group, in turn, had higher values than the control group (*P* < 0.05). These results suggest that CHA/SF/GCS/DF-PEG hydrogel composites can promote bone tissue regeneration and the loading of hucMSC-derived exosomes can enhance the effect of hydrogel bone formation.

#### H&E Staining Results

**FIGURE 7:**
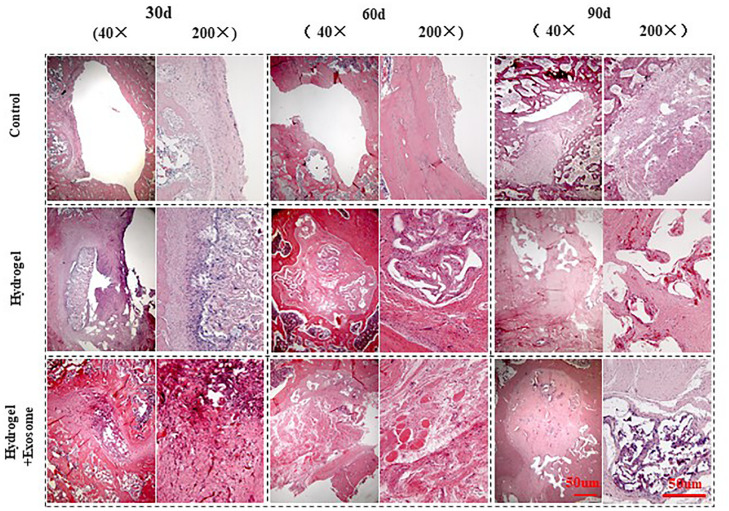
H&E staining images of bone defect sections after 30, 60 and 90 days post-implantation of CHA/SF/GCS/DF-PEG hydrogel or CHA/SF/GCS/DF-PEG hydrogel associated with hucMSC-derived exosomes (40× and 200× magnifications).

H&E staining pictures of bone defect repair show that no apparent immune cells or inflammatory cell aggregations were seen in any of the groups analyzed (control or experimental) 30, 60, and 90 days post-implantation of CHA/SF/GCS/DF-PEG hydrogel or CHA/SF/GCS/DF-PEG hydrogel associated with hucMSC-derived exosomes (Figure 7). In addition, pathological features such as infection, necrosis and massive bleeding were not observed. These data prove that the composites of both hydrogel preparations have good histocompatibility and do not cause immunological rejection.

The control group showed visible bone defect areas at 30, 60, and 90 days after implanting the hydrogel preparations compared to the two experimental groups. In addition, a thin layer of fibrous connective tissue was observed covering the area around the bone defect. Finally, in the control group, no new characteristic bone tissues or apparent infiltration of osteoblasts or chondrocytes were observed Therefore, this group had a weak repair effect. In the hydrogel and hydrogel-exosomes experimental groups, the bone graft material was totally degraded after 90 days of implantation and only a small amount of CHA was spread in the interstitial space. In the hydrogel group, a large number of osteoblasts infiltrated along the porous scaffold was observed after 30 days of implantation. In addition, the spread of new bone tissue was observed, as well as a small amount of new blood vessels. Therefore, the bone defect in hydrogel group was significantly less than in the control group. Also in the hydrogel group, after 60 and 90 days of the implantation, the bone defect was almost filled and the new bone tissue was tightly fused to each other in a honeycomb shape. A large number of mature vascular networks can be also observed in the pores of incompletely degraded CHA, as well as a small amount of new bone. In contrast, in the hydrogel-exosomes group, after 30 days of implantation, a large number of osteoblasts and vascular networks were seen already observed and the materials were already tightly bound to the new bone tissue. After 60 and 90 days, besides a large number of osteoblasts and an abundant extracellular matrix, a new thick and regular bone tissue was observed. Moreover, the edges of the residual bone defect area have become dull and the defect area has become dense. The new bone tissue was filled with a large number of new blood vessels that had a large number of chondrocytes and mature bone cells around them.

#### Masson’s Trichrome Staining Results

**FIGURE 8:**
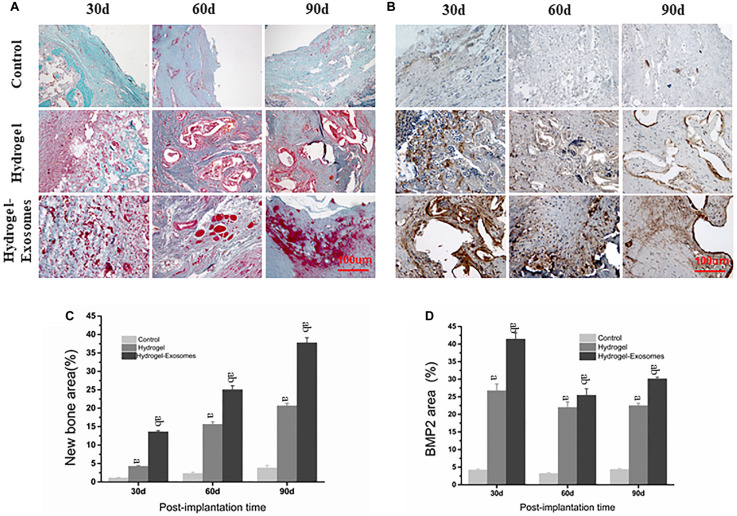
Masson’s trichrome and BMP-2 staining of bone defect sections and quantitative analysis of bone deposition 30, 60 and 90 days post-implantation of CHA/SF/GCS/DF-PEG hydrogel or CHA/SF/GCS/DF-PEG hydrogel associated with hucMSC-derived exosomes. Representative images of Masson’s trichrome staining **(A)** and BMP-2 staining **(B)** of the bone defect sections. In Masson’s trichrome staining, the new bone tissue is observed in red and the collagen tissue in blue-green. Quantitative analysis of the percentage of new bone area formed **(C)** and BMP-2 stained area **(D)** in the control and in the two experimental groups in the three observed times. a, *P* < 0.05 compared to the control group; b, *P* < 0.05 compared to the hydrogel group.

Masson’s trichrome staining images highlighted the results obtained previously by H&E staining image analysis, most prominently displaying the tissue microstructure and extracellular matrix components (Figure 8A). In the control group, after 30 and 60 days of implantation of the hydrogel preparations, only a small amount of disorderly fibrous connective tissue was observed. A small amount of new bone tissue was observed scattered only after 90 days of application. In the hydrogel group, a large number of osteoblasts filled the empty spaces of the hydrogel material 30 days after implantation and some bone repair structures appeared scattered after 60 and 90 days. The number of irregular organizations observed was low. In the hydrogel-exosomes group, bone repair was the most evident. After 30 days, bone repair structures were already very visible around blood vessels and osteoblasts. After 60 and 90 days, large and regular new bone tissues, with peaking and nesting, were observed and the collagen also matured. Data analysis found that the bone content of the hydrogel group was significantly higher than that of the control group (*P* < 0.05). In addition, the hydrogel-exosomes group had significantly higher bone content and more regular bone morphology than the other two groups (*P* < 0.05) (Figures 8A,C).

#### BMP-2 Staining Results

BMP-2 staining images show that BMP-2 is highly expressed in the material pores and its surrounding areas (Figure 8B). These data indicate the occurrence of an active osteogenesis process, which is in line with the characteristic of BMP-2 in being easy distributed around the porous structure. The peak expression of BMP-2 was expressed 30 days after implantation of the hydrogel preparations. After 30, 60, and 90 days of this implantation, BMP-2 expression in the hydrogel-exosomes group was higher than in the hydrogel group, which in turn showed a significantly higher BMP-2 expression than the control group (*P* < 0.05) (Figures 8B,D).

#### hucMSC-Derived Exosomes Promote HUVECs Proliferation

**FIGURE 9:**
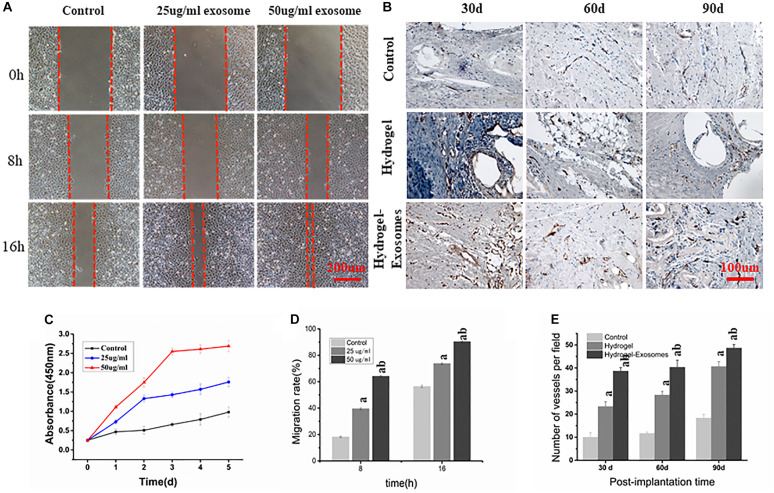
**(A)** Scratch Wound Assay (Scale bar: 200 μm). **(B)** Representative images of immunohistochemical CD34 staining 30, 60 and 90 days post-implantation of CHA/SF/GCS/DF-PEG hydrogel or CHA/SF/GCS/DF-PEG hydrogel associated with hucMSC-derived exosomes (Scale bar: 100 μm). **(C)** Effect of hucMSC-derived exosomes on the proliferation of HUVECs. **(D)** Migration rate of HUVECs observed in scratch wound assay in the presence of 0 (control), 25 and 50 μg/ml of hucMSC-derived exosomes. **(E)** Quantitative analysis of the number of microvessels per field 30, 60 and 90 days post-implantation of the two hydrogel preparations. a: *P* < 0.05 vs. Control, b: *P* < 0.05 vs. 25 μg/ml exo or hydrogel.

CCK-8 measurement results showed that, compared to the control group, the proliferation of HUVECs in the experimental groups that contained 25 and 50 μg/ml of hucMSC-derived exosomes increased significantly after 1, 2, 3, 4, and 5 days of culture (*P* < 0.05). Furthermore, as the concentration of exosomes increased, proliferation became more pronounced, showing a dose-dependent effect (Figure 9C). These results show that hucMSC-derived exosomes can promote the proliferation of HUVECs.

#### Effect of hucMSC-Derived Exosomes on HUVECs Migration

Scratch wound assay results showed that hucMSC-derived exosomes can promote the migration of HUVECs to the blank area. After 8 h, the groups containing 25 and 50 μg/ml hucMSC-Derived exosomes had a significantly higher HUVECs migration rate than the control group (39.57% ± 3.63%, 68.57% ± 3.35%, and 19.94% ± 2.78%, respectively. *P* < 0.05). After 16 h, the HUVECs mobility observed in groups containing 25 and 50 μg/m exosomes was still greater than that of the control group (75.47% ± 4.21%, 90.76% ± 3.69%, and 57.92% ± 3.58%, respectively; *P* < 0.05). These data also show that HUVECs mobility in the 50 μg/ml exosome containing group was higher than that of the 25 μg/ml exosome containing group (*P* < 0.05) (Figures 9A,D).

#### Immunohistochemical Analysis of Microvessel Density

CD34 immunohistochemical staining was performed 30, 60, and 90 days post-implantation of CHA/SF/GCS/DF-PEG hydrogel or CHA/SF/GCS/DF-PEG hydrogel associated with hucMSC-derived exosomes to analyze angiogenesis in the new bone (Figures 9B,E). More newly generated microvessels were observed in the hydrogel-exosomes group 30 days after implantation. After 60 and 90 days, the blood vessels in this group matured and were associated with typical round or oval microvessels (Figure 9B). Quantitative analysis of microvessels showed that, for all groups studied (control and two hydrogel preparations), the number of microvessels increased when analyzed 30 and 90 days after implantation. Moreover, in the hydrogel-exosomes group, the number of microvessels was significantly higher than that in the hydrogel group, which had a significantly higher number of microvessels than the control group (*P* < 0.05, Figure 9E).

## Discussion

The treatment of bone defect remains an ongoing challenge for the orthopedic surgeon. Remarkably, bone grafts are widely used because they fill the empty spaces and provide support for bone defect corrections, thereby facilitating their biological repair (Marecek et al., 2020). In the present study, we were able to manufacture a CHA/SF/GCS/DF-PEG hydrogel associated with hucMSC-derived exosomes that was able to promote bone defect healing.

Throughout human life, bones are always in the delicate dynamic balance of synthesis and absorption through the cooperation or symphony of various cell types of osteoclasts, osteoblasts, vascular endothelial cells and their precursors to achieve fine bone minerals metabolism process (Tao and Guo, 2019). The cell-to-cell communication for the coordination of bone remodeling occurs in part through exosomal exchange. Steering the MSCs towards or away from osteoblastic differentiation is pivotal in this regard (Cui et al., 2016; Qin et al., 2016). In addition, exosomes, as the primary biological component of the paracrine function of stem cells, reduce the possibility of stem cells forming tumors in the body due to their inability to divide and differentiate. Thus, exosomes are relatively more stable than cell transplantation therapy (Marbán, 2018). Interestingly, exosomes relevant to bone remodeling are secreted not only dominated by the main role of bone physiology but also by various other cell types, such as synovial fibroblasts (Kato et al., 2014), adipocytes (Martin et al., 2015), dendritic cells (Silva et al., 2017), the endothelium (Weilner et al., 2016), myoblasts (Xu et al., 2018), and hucMSC (Li et al., 2019; Yang et al., 2020).

HucMSCs are derived from postpartum waste tissues, rich in sources and their use is devoid of ethical and moral disputes. Compared to other tissue sources, hucMSCs are derived from humans, have no species differences and have more enormous clinical potential. In this study, the widely known low-temperature ultracentrifugation method was used to obtain pure hucMSC-derived exosomes. These exosomes were cup-shaped under the electron microscope and were mostly ∼90 nm in size. Western blot assays showed that the obtained hucMSC-derived exosomes were able to express the surface proteins characteristic CD9 and CD63, indicating that they were successfully isolated. *In vitro* experiments we used hucMSC-derived exosomes to stimulate mOPC and HUVECs. We found that hucMSC-drived exosomes have osteogenicity capacity, because they can improve the osteogenic differentiation ability of mOPCs, as well as the proliferation and migration of HUVECs in a concentration-dependent manner, which may be related to hucMSC derived exosomes as an intercellular communicator upregulating HIF-1α (Zhang et al., 2019) and controlling BMP-2 and/or VEGF gene expression of target cells (Li et al., 2019), may be one of the underlying mechanisms in the promoted process.

Some studies have reported that no significant effect was observed with free exosomes treatment, because of its rapid excretion from the of site of application (Zhang Z. et al., 2018; Wang et al., 2019). It is likely that free exosomes diffused out from the defect rapidly, resulting in no exertion of growth factor activity (Riau et al., 2019). In addition, there is no substance present to prevent the infiltration of soft tissue into the bone defect, which suppresses regeneration of bone tissue (Hokugo et al., 2005). In the selection process of the exosome application carrier, we comprehensively considered the characteristics of the bone graft material. In this way and in accordance with the biomimetics concept, CHA was used as the main component of bone graft material, filling the entire hydrogel material to simulate the inorganic part of natural bone and maintain the balance of calcium and phosphate in the defect area. The SF has a structure similar to that of skeletal collagen, is conducive to calcium salt deposition and has no antigenicity. GCS is a polycationic polysaccharide composed of glucosamine and *N*-acetylglucosamine residues that can mimic extracellular matrix components *in vivo* and act as a substrate for cell adhesion (Bharadwaz and Jayasuriya, 2020). The GSC has multiple acetylamino groups on its molecular backbone and can react with the gel factor DF-PEG as cross-linking sites to form a dynamic and uniform three-dimensional mesh structure. The network crosslinking point of a hydrogel containing GSC and DF-PEG is an imine bond (also known as a Schiff base bond) (Yang et al., 2012). Due to the inherent dynamic balance between the imine bond and the aldehyde and amine reactants, the interaction can be considered pseudo-covalent. These properties indicate that a GSC/DF-PEG hydrogel could have a high self-healing ability. In the present work, we were able to successfully prepare a CHA/SF/GCS/DF-PEG hydrogel in 60 s at room temperature by simple stirring. This hydrogel can effectively prevent rapid changes in environmental conditions during the gelation process and can be restored on its own after several incisions, indication a good self-healing ability. Moreover, we showed that the CHA/SF/GCS/DF-PEG hydrogel has good biocompatibility to be used as bone graft material. MTT assays revealed that CHA/SF/GCS/DF-PEG hydrogel is not cytotoxic for mOPCs. In addition, morphological observations of three-dimensional cultures showed that mOPCs presented good growth in the CHA/SF/GCS/DF-PEG hydrogel. Finally, LIVE/DEAD staining revealed that the survival rate of mOPSc on the 3^rd^ of culture in the hydrogel was over 90%.

The effectiveness of commercial bone graft materials is known to be hampered by the poor efficiency of cell growth. Xu et al. (2020) found that the macro pore forming strategy, especially bioactive macropores, has a good potential to circumvent this problem and thus promote fracture healing. SEM image analysis revealed that the CHA/SF/GCS/DF-PEG hydrogel has a uniform pore diameter, high porosity and excellent connectivity between pores. Besides, this hydrogel has excellent hydrophilicity compared to conventional gelatin hydrogels. Good hydrophilicity is favorable for the adsorption of water-soluble proteins on the hydrogel surface and guarantees suitable adhesion to cell. In the experiment, we can observe by Masson staining that at 30 days, the filled hydrogel in the defect area was not completely degraded, showing a blue-stained mesh distribution. In addition, it was possible to observe that bone-derived mesenchymal stromal cells grow and migrate along the CHA/SF/GCS/DF-PEG hydrogel scaffold and differentiate into bone cells. However, the Masson staining results at 60 days showed that the materials except CHA were almost completely degraded.

Inorganic phosphate (Pi) is necessary for cell metabolism and signal transduction and is also an indispensable structural component of the extracellular matrix (Chande and Bergwitz, 2018). In this study, we selected horn corals with a pore size of about 100 μm. Subsequently, we obtained CHA by replacing the carbonate from the natural calcium carbonate of the corals with phosphate. This replacement was performed by hydrothermal reaction in the presence of diammonium hydrogen phosphate (Georgiannos et al., 2015). The calcium and phosphorus components of CHA are degraded slowly degraded in the body so that their concentrations remain constant during bone tissue repair. The inorganic component of CHA has an architecture similar to human bone while maintaining the natural porous structure of corals. Previous studies have shown that specific surfaces of porous hydroxyapatite ceramics do support osteoblastic cell differentiation and the expression of the osteoblastic phenotype (Akahane et al., 1993; Klar et al., 2009). Over time, osteoblasts gradually enter the CHA scaffold, differentiate into osteocytes that grown in the hydroxyapatite micro gap of hydroxyapatite. Osteocytes gradually absorb hydroxyapatite degradation products and replace implant materials to restore the typical bone structure. Therefore, CHA plays an essential role in the reconstruction of subchondral bone. At 90 days, the micro-CT detection could clearly observe the incompletely degraded CHA white highlight images, suggested that the experimental group still had a certain osteogenic effect at 90 days.

Here, using CD34 and BMP2 immunohistochemical staining methods, we highlighted the major biological activities of CHA for bone repair. Its porous structure provided scaffolds for the invasion of blood vessels and the formation of new bone, thus promoting osteogenesis. In addition, the porous surface promoted the aggregation of BMP-2. BMP2 has osteoinductive properties, which is the main driving force for recruiting stem cells and inducing angiogenesis and osteogenesis, further enhancing the osteoinductive effect of CHA (Yao et al., 2017). CHA/GCS/SF/DF-PEG hydrogel is a promising scaffold with which to deliver exosomes growth factors to bone defects and to assist bone regeneration at such defects by physically preventing soft tissue infiltration and in biological activity aspect through stimulating BMP2 deposition and angiogenesis. However, we believe that our experiments also have some limitations. First, the animal experiment design process lacked a control group to prove the advantages of CHA/GCS/SF/DF-PEG hydrogel over other bone transplantation materials. Second, we believe that if the effect of hydrogel osteogenesis can be further demonstrated by *in vitro* cell experiments, may give our results more arguments. Third, the exact mechanism of osteogenesis of CHA/GCS/SF/DF-PEG carrying hucMSC-derived exosomes is unknown and needs further study.

## Conclusion

In conclusion, we have presented an inexpensive, simple, and rapid method to prepare dynamic hydrogels using CHA/GCS/SF and DF-PEG as main components. The hydrogel has desirable structural and physical properties that can be beneficial for bone healing and can be used as a scaffold for the exosomes. The combination of the exosomes and hydrogel could effectively promote the bone healing in SD rat model by promoting the BMP2 deposition, bone collagen deposition and maturation and enhancing angiogenesis. This study will hopefully provide a solution for bone defect healing in clinical practice and provide a scientific basis for the achievement of further cell-free therapy.

## Data Availability Statement

All datasets generated for this study are included in the article/supplementary material.

## Ethics Statement

The animal study was reviewed and approved by the Department of Advanced Interdisciplinary Studies, Institute of Basic Medical Sciences and Tissue Engineering Research Center, Academy of Military Medical Sciences, Beijing, China.

## Author Contributions

LW and JW: experimental work performance and manuscript drafting. XZ, JS, and PC: data collection and related analysis. BZ, XG, CD, and TZ: data analysis and manuscript revision. HG, TZ, and XG: study design and coordinating experiment. All authors read and approved the final manuscript.

## Conflict of Interest

The authors declare that the research was conducted in the absence of any commercial or financial relationships that could be construed as a potential conflict of interest.
